# A Multi-Sensor Matched Filter Approach to Robust Segmentation of Assisted Gait

**DOI:** 10.3390/s18092970

**Published:** 2018-09-06

**Authors:** Satinder Gill, Nitin Seth, Erik Scheme

**Affiliations:** 1 Institute of Biomedical Engineering, University of New Brunswick, Fredericton, NB E3B 5A3, Canada; satinder.gill@unb.ca (S.G.); nitin.seth@unb.ca (N.S.); 2 Department of Electrical and Computer Engineering, University of New Brunswick, Fredericton, NB E3B 5A3, Canada

**Keywords:** multi-sensor, assistive device, cane, gait analysis, loading information, inertial measurement unit (IMU), stride segmentation

## Abstract

Individuals with mobility impairments related to age, injury, or disease, often require the help of an assistive device (AD) such as a cane to ambulate, increase safety, and improve overall stability. Instrumenting these devices has been proposed as a non-invasive way to proactively monitor an individual’s reliance on the AD while also obtaining information about behaviors and changes in gait. A critical first step in the analysis of these data, however, is the accurate processing and segmentation of the sensor data to extract relevant gait information. In this paper, we present a highly accurate multi-sensor-based gait segmentation algorithm that is robust to a variety of walking conditions using an AD. A matched filtering approach based on loading information is used in conjunction with an angular rate reversal and peak detection technique, to identify important gait events. The algorithm is tested over a variety of terrains using a hybrid sensorized cane, capable of measuring loading, mobility, and stability information. The reliability and accuracy of the proposed multi-sensor matched filter (MSMF) algorithm is compared with variations of the commonly employed gyroscope peak detection (GPD) algorithm. Results of an experiment with a group of 30 healthy participants walking over various terrains demonstrated the ability of the proposed segmentation algorithm to reliably and accurately segment gait events.

## 1. Introduction

Studies have shown that one out of every three American adults aged 65 and older falls each year, often resulting in serious injuries and loss of independence [1]. The related surgeries, rehabilitation and recovery times result in the prolonged use of health care services contributing substantially to high and rising health care costs [2,3]. With a globally aging population, and the baby-boomer population entering retirement, projected fall rates and healthcare spending are projected to increase unsustainably [4,5]. In order to combat these growing costs, a growing emphasis has been placed on monitoring solutions capable of identifying changes in gait and stability prior to falls, facilitating early intervention and preventing added burden on healthcare system [6,7,8,9]. 

Proactive monitoring of gait can not only enable early interventions to help prevent falls, but it can also provide insights into the health and behaviors of an individual [7,10,11,12]. As this necessitates observation of behaviors of everyday life, as opposed to controlled assessments in a lab or clinic with a specialist, recent trends in gait research have focused on enabling continuous monitoring in the community [13,14,15]. This is often promoted using either wearable devices or using existing items that have been specially instrumented [14,16,17,18,19,20,21,22,23].

Traditionally, researchers have used kinematic and kinetic-based equipment, such as optical motion capture systems and force plates, in controlled laboratory settings. These systems are considered to be the gold standard in gait monitoring, but also have associated limitations [13,19,24,25]. In addition to being cost prohibitive for most clinical use, these systems cannot easily be used in real-life settings outside of a laboratory. As such, their use is generally restricted to fixed environments, enabling the capture of only a small number of consecutive gait cycles. Combined with the need for highly skilled personnel to operate and analyze the data, these systems are not a viable option for community-based monitoring.

Wearable sensor systems, enabled by smartphones, foot switches, pressure insoles, accelerometers, and gyroscopes present a more portable, low cost, and easy to use alternative to traditional motion capture systems. Consequently, wearable sensor systems have gained substantial interest in the research community for monitoring gait activity [16,17,26,27,28,29,30,31]. Given their ubiquity in today’s society, the accelerometers within mobile phones have been widely explored for recognition of gait and walking terrain [32,33,34]. Gyroscopes have also been widely used for gait tracking due to their low cost and ease of use and immunity to vibrations caused due to heel strike [17,35,36,37]. While such wearable technologies are better suited for use outside of clinical and laboratory settings, however, they have yet to find widespread adoption among the populations that could most benefit from their use [38]. In particular, concerns among seniors and vulnerable patient groups include a lack of technological knowledge, cognitive ability, obtrusiveness of the devices, an opposition to behavioral change, and a lack of demonstrated value in a healthcare setting [23].

One solution to address some of these challenges is to integrate information-gathering elements into the regular everyday objects already being used by this population. It is estimated that currently 6.1 million community-dwelling American adults ambulate with the support of an assistive device (AD), such as a cane or walker, to combat mobility impairments and mitigate instability [39]. This has led researchers to develop instrumented ADs to help monitor and categorize gait activity [4,40,41]. In particular, both walkers and instrumented canes have been proposed [39,40,42,43]. While these early works demonstrated the potential of this approach, most of these initial prototypes have lacked the industrial design needed for deployment outside of training situations, or used expensive components such as load cells that could limit their widespread deployment [18]. 

In our prior work [18], we presented the design of a multi-sensor, Internet-of-Things (IoT) enabled cane, which is nearly indistinguishable from a traditional offset cane. That work focused on the development of a robust, affordable, and passive means of gait monitoring by improving upon previous devices in technical and industrial design. Strain gauges were used to capture loading information more reliability than with FSRs and at a lower cost than load cells [4,41]. It was also demonstrated that this loading information, combined with angular velocity data, could be used to reliably detect the initial contact (IC) and terminal contact (TC) events of the gait cycle.

Regardless of the measurement system used, the reliability and accuracy of gait segmentation directly influence the quality of further analyses and inferences. In the literature, a number of different algorithmic techniques have been applied to the gait segmentation problem. Peak-detection with thresholding is the most common approach for gait segmentation, especially for studies that use IMU data [40,44,45]. Although popular due to their simplicity, thresholding approaches are often challenged when handling noisy data or outliers [14,19,44]. Ultimately, however, it is hope that these types of sensors will become widely adopted in the community, leading to a much greater variability of usage scenarios. One simple example of this increased variability may result from walking on terrains other than flat ground, or when transitioning from a flat terrains to ramps or stairs [46].

To handle these more variable situations, more complex techniques have been applied. Probabilistic models, such as Hidden Markov Models (HMMs), are capable of handling uncertainty with dynamical models and have demonstrated the ability to recognize stride segments and activities [19,47]. These techniques, however, require the collection and determination of data quantiles [19] as well as prior and posterior probabilities which introduce further complexity, computational requirements, and assumptions to the system. To maximize generality and minimize computational complexity, novel, simple techniques are warranted.

Alternatively, signal processing techniques that exploit the temporal structure of the signal have been proposed for instrumented gait segmentation [6,48,49]. In particular, matched filtering [45,50] has been shown to effectively exploit the periodicity of the signal for gait segmentation while adding minimal complexity to the segmentation algorithm. Such approaches offer advantages over amplitude-based thresholding techniques, but have been shown to experience issues when the temporal structure varies, such as with varying stride durations [44]. Variable stride durations, however, are common in real-life walking conditions and in clinical settings, such as during rehabilitation from spinal cord injury or therapy for Parkinson ’s disease [14,51].

This presents a significant challenge for gait segmentation and analysis in every-day situations when ambulation is seldom continuous. In the literature, however, studies have often been restricted to flat terrain in controlled lab conditions. Even under these ideal conditions, force plates have been used to confirm that gyroscope-based methods introduce errors when segmenting gait according to IC and TC gait events [13,24,50]. 

In this work, we present a multi-sensor based form of gait segmentation that is applicable to a variety of walking terrains and transitions between them. Segmentation accuracy across different walking terrains is compared for subjects walking with an instrumented cane. The experimental results are presented to validate the proposed segmentation algorithm for various walking terrains.

## 2. Materials and Methods

### 2.1. System Overview

**Figure 1:**
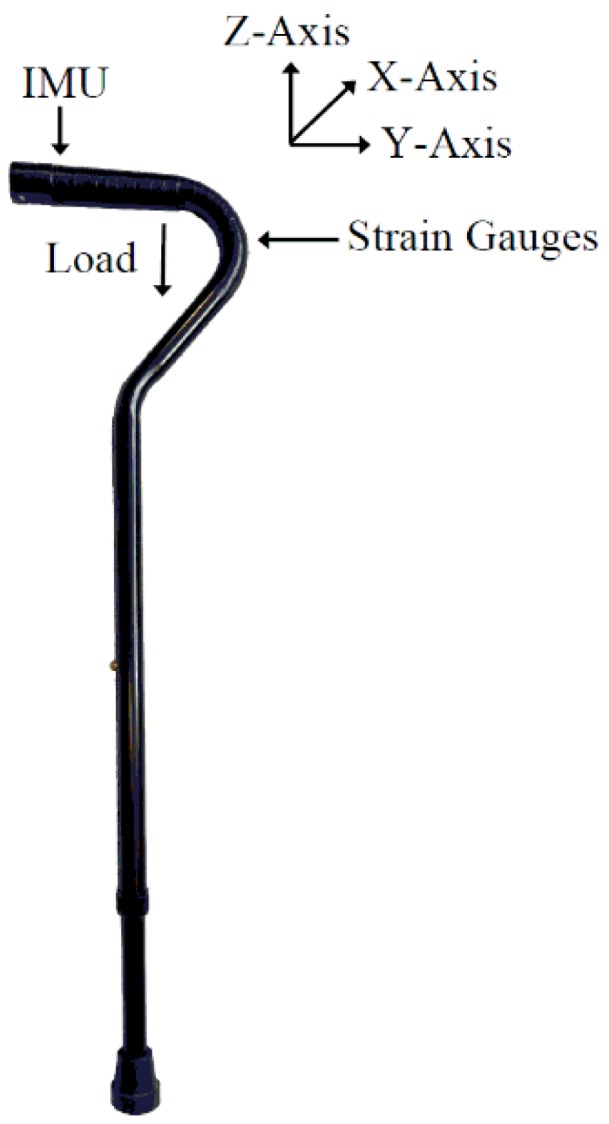
IoT-enabled cane with electronic design implemented within the handle of the cane, as described in [18].

A multi-sensor IoT enabled cane described in detail in [18], consisted of a inertial measurement unit (IMU) and strain gauges affixed along the curvature of the cane. The SGT-1A/1000-TY13 (Omega Engineering, Stamford, CT, USA) strain gauges, arranged in Wheatstone topology, were used to provide loading information during cane use. The LSM9DS1, a 9-axis IMU, provides accelerometer, gyroscope and magnetometer readings. The IMU was set to provide angular velocity with a sensitivity of 500 degrees/s and acceleration with a sensitivity of 16 g. The IMU and strain gauges are sampled at 231 Hz. Figure 1 shows a picture of the final design of the cane with all electronics implemented within the handle of the cane. Two coordinate systems were used: the IMU coordinate system and a global Cartesian system. The global Cartesian system is a fixed reference system with the z-axis of the global Cartesian system in parallel with the gravitational acceleration. The XY plane is assumed to be level and perpendicular with the y-axis pointing in the walking direction. The three axes of the IMU coordinate system coincided with the three axes of the global Cartesian system. 

### 2.2. Experimental Protocol

A total of thirty healthy participants were recruited from the local community (24 male and six female, aged 18–31 years, mean ± SD = 22.0 ± 3.1 years) and gave informed written consent as approved by the University of New Brunswick Research Ethics Board (REB #2017-097). Participants were given a brief tutorial on cane-assisted gait and a familiarization session where they could walk freely with the instrumented cane. Cane height was adjusted for each subject based on [52]. Once the participant and examiner agreed that the participant was sufficiently comfortable using the cane and could engage in conversation without affecting their gait, the experiment and data collection proceeded. Each participant was instructed to walk with the help of the cane at a self-selected speed over a variety of terrains, as listed in Table 1. Two-minute breaks were taken between each scenario to allow for rest and instructions for the next terrain.

### 2.3. Data Analysis

**Figure 2:**
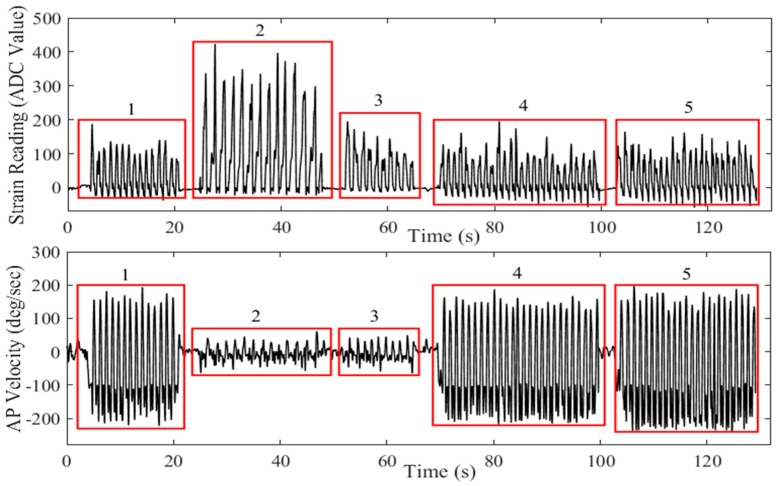
Strain gauge and gyroscope AP velocity readings for different walking terrains. 1—Flat surface, 2—Upstairs, 3—Downstairs, 4—Uphill, 5—Downhill.

The raw data for each participant was collected and stored for offline processing. High-frequency noise components were removed from the strain gauge and IMU data using sixth-order, low-pass filters with 4 and 8 Hz cutoff frequencies, respectively. Data analysis was carried out using MATLAB^®^. Figure 2 shows an example of data recorded from the strain gauges and gyroscope AP velocity for different terrains. Data presented here was taken from an example participant while walking across various terrains and has been concatenated to highlight the differences in sensor values for various conditions.

To demonstrate the reliability, accuracy, and robustness of the proposed multi-sensor matched filter (MSMF) algorithm, its ability to segment strides was compared with variations of the commonly employed gyroscope based peak detection (GPD) algorithm [40]. The GPD algorithm was tested for several different thresholds: GPD-O, GPD-IFS, GPD-UFS, and lastly, GPD-U, as outlined in Table 2.

It should be noted that the GPD-O is included only as an ideal baseline comparison, as it is unlikely to be practical during regular use given that the terrain would first have to be classified prior to segmentation. Paired sample t-tests using an alpha value of 0.05 were performed to compare the true and false positives; *p* values less than 0.001 are represented using *p* < 0.001.

## 3. Gait Segmentation and Assessment

### 3.1. Multi-Sensor Fusion Gait Segmentation Algorithm

**Figure 3:**
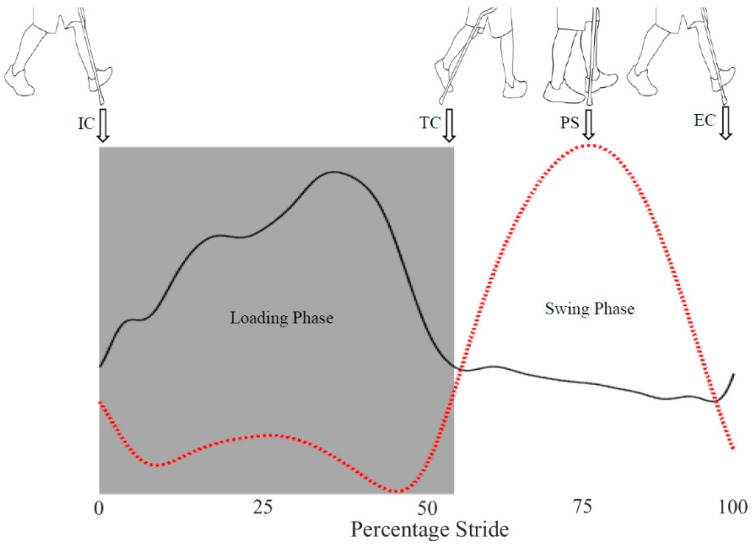
IC, TC and EC events corresponding to strain gauge data (solid black line); PS event corresponding to peaks in gyroscope AP velocity (dotted red line).

Gait segmentation was used to identify individual strides and detect relevant walking cycle events such as initial-contact (IC), terminal-contact (TC), peak-swing (PS), and end-contact (EC) events. The algorithm was developed by categorizing the walking cycle into two phases: the cane loading phase and the cane swing phase. The loading phase starts with the IC event, when the user extends the cane forward to touch the ground before taking a step and loading the cane for support while moving forward. The loading phase ends with the TC event, when the user stops loading the cane and gets begins to lift the cane of the ground. The swing phase follows, during which the user swings the cane in anteroposterior (AP) direction to relocate the tip of the cane for next loading phase. The moment during the swing phase when the gyroscope AP velocity reaches a maximum is identified as the PS event. The swing phase ends with the EC event when the user stops swinging the cane and again places the tip of the cane in front of them. In this way, a gait cycle is defined as the interval between the IC and EC events the cane. An example of this is presented in Figure 3 where IC and TC events correspond to positive and negative zero-crossings in the strain gauge data, respectively, whereas the PS event corresponds to a peak in the gyroscope anteroposterior (AP) velocity. The EC event is also shown corresponding to the strain gauge data. For a continuous walking scenario, the EC event also corresponds to IC event of the next stride.

A matched filtering approach was used to detect these events of interest from the different data sources. A matched filter is an optimal linear filter, which works by correlating a known template signal with the unknown data to gauge the likelihood of the presence of the template signal within the unknown data. Consider the template signal, h(t), and an unknown signal as x(t). The output of the matched filter is represented by the following equation:

(1) $$r\left(\tau \right)=\int_{-\infty }^{\infty }h\left(t\right)\;x\left(t+\tau \right)\;dt$$

**Figure 4:**
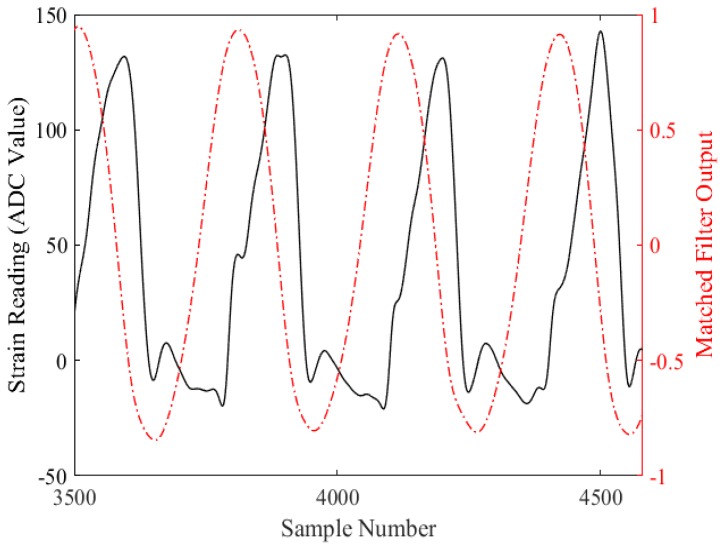
The matched filter output (dotted red line) obtained when correlating the template signal with unknown strain gauge data (solid black line).

Figure 4 shows the matched filter output obtained by correlating the known template signal with a selection of unknown data from a participant. As can be seen, the matched filter is able to output positive and negative peaks corresponding to occurrence of IC and TC events, respectively.

**Figure 5:**
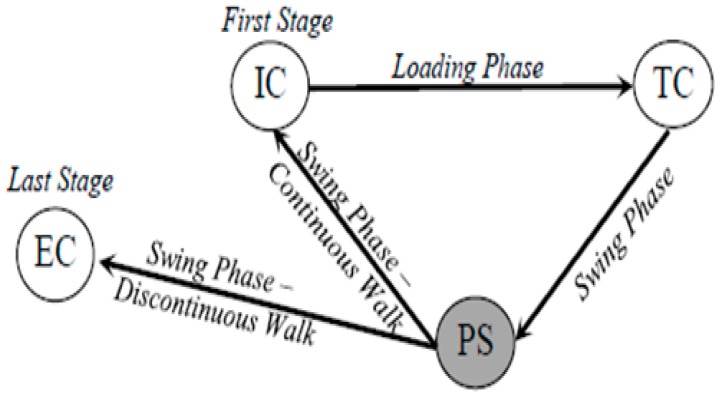
State machine diagram outlining algorithm logic for a single stride. Algorithm starts by detecting the first IC point when the cane strikes the ground.

Figure 5 shows the state machine used for this approach. A matched filter was used to identify the starting state (i.e., IC event) by correlating a known template signal with the unknown strain-gauge data to detect the presence of the template pattern within the data [50]. The template signal was selected at random from a pilot participant’s strain gauge data corresponding to a single stride during flat surface walking (similar to that presented in Figure 3), and was used for all of the varying terrains for all participants. The matched filter outputs distinct positive peaks at instances of IC events. To correctly detect the presence of this template signal within the unknown data, a similarity threshold of 50% was used. Any spurious positive peaks in the matched filter output that showed less than 50% correlation were therefore discarded and not counted as a stride. Identification of a valid positive peak in the matched filter output thus indicated the presence of the template signal in the data. It should be noted that this threshold is unlike the commonly used amplitude threshold, as it is a representation of the shape of the temporal signal, and not dependent on a particular value.

To account for the varying stride lengths, positive strain gauge zero-crossing points (i.e., strain gauge transitions from negative to positive) within 0.5 s of the matched filter’s positive peaks were identified as IC events. Similarly, negative strain gauge zero-crossing points (i.e., strain gauge data transitioning from positive to negative) following the IC event were used to identify the next state (i.e., TC event). Identification of IC and TC events validate the existence of loading phase of a gait cycle.

Next, the gyroscope AP velocity was used to indicate the presence of the swing phase. AP velocity peak detection was used in conjunction with the angular rate reversals, which can be reliably and accurately identified regardless of gait pattern [25], for identification of the swing phase. The existence of a peak gyroscope AP velocity following a TC event and negative-to-positive rate reversal (initiation of forward swing) in the AP velocity indicated the existence of a PS event in the gait cycle.

**Figure 6:**
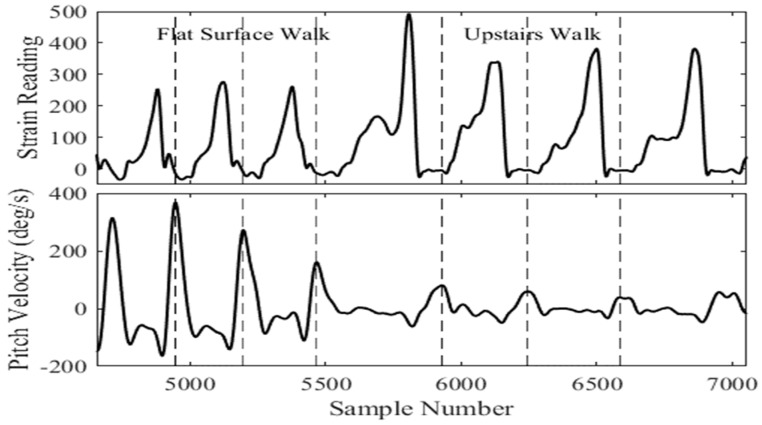
Changes in strain gauge data (above) and gyroscope AP velocity (below) when transitioning from flat surface walking to upstairs walking. Dotted lines indicate the location of peak swing.

Unlike conventional methods, no amplitude threshold value is used for peak identification. This is important as the gyroscope AP velocity amplitude can change significantly while walking across different terrains. This, in effect, compromises the robustness of existing algorithms to different terrain scenarios. An example of this is shown in Figure 6 (bottom) where a substantial change in amplitude of the AP velocity occurs when transitioning from flat terrain to walking up stairs. Additionally, the peak AP velocity doesn’t necessarily correspond to the mid-point in the swing phase for conditions such as walking up or down stairs. Therefore, the term Peak Swing or “PS event” is used in this paper instead of the term “MS event” generally used in the gait literature [40,50].

Once the PS event is identified, the next state corresponds to the end of a walking cycle, here named the End Contact (EC) event. The EC is identified as a first point of contact the cane makes with the ground following the swing phase. During continuous walking, this event corresponds to the IC event of the next gait cycle. Its inclusion, however, allows for the assessment of intermittent gait or final strides before stopping. Each valid gait cycle is identified by the existence of consecutive TC, PS and EC events within 3 s of an IC event. In this fashion, all valid gait cycles and corresponding temporal parameters (i.e., IC, TC, PS and EC events) of the walking cycle are identified.

### 3.2. Algorithm Evaluation

**Figure 7:**
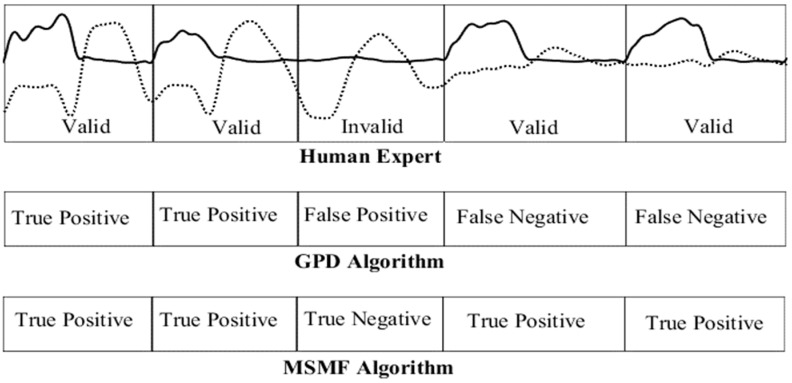
Stride classification by human expert (top) and GPD algorithm (middle) and MSMF algorithm (bottom) as participant transitions from walking on a flat surface (first two segments) to walking on stairs (final two segments). Solid lines indicate strain gauge data and dotted line indicates gyroscope AP velocity.

The detection of valid gait cycles obtained using the MSMF and GPD algorithms were compared with results obtained by manual inspection of a human expert [40,53]. For manual inspection, a valid gait cycle was defined as a gait cycle consisting of a loading phase (weight being exerted on the cane) followed by a swing phase (no weight being exerted on the cane while swinging the cane in the AP plane). All other gait cycles (i.e., no load being exerted on the cane while swinging the cane, load being exerted on the cane while not swinging the cane) were listed as invalid gait cycles. Four categories of stride identification performance were identified and assessed: true positive (gait cycled identified by both the algorithm and human expert), true negative (gait cycle rejected by human expert and by algorithm), false negative (gait cycle identified by human expert but missed by algorithm), and false positive (gait cycle identified by algorithm but rejected by human expert). Figure 7 shows an example of each case, classified by the human expert, GPD, and MSMF algorithms.

A comparison of the variance of stride lengths obtained using MSMF and GPD algorithms is also used to evaluate the algorithm performance. Stride variability was calculated as the variance of detected peak-swing events, for each participant, for each terrain. This was included because the variance of the stride lengths increases when an algorithm detects false positive or false negative. As such, an accurate algorithm should yield the lowest possible variance, indicative of the user’s true stride variability.

### 3.3. System Verification (Pilot)

In addition to stride classification and variability analyses, an additional pilot study was conducted to evaluate the accuracy of the algorithms in capturing temporal gait cycle events. For this purpose, a push-switch was integrated into the bottom of the cane developed in [18] in order to provide discrete information corresponding to cane-floor contact and lift-off events. The binary push-switch input (i.e., close vs. open) was used to identify IC and TC events. Data was collected with the push-switch for 5 participants of the 30 participants as a proof of concept, and these data were incorporated into the full experimental procedure. This push-switch was not incorporated into the actual device design due to its lack of robustness and the susceptibility to wear and tear at the end of the cane. For fair comparison, only events where both the MSMF and GPD algorithms successfully identified a valid stride were considered in the analysis with the push-switch. 

## 4. Results

### 4.1. Stride Variability

**Figure 8:**
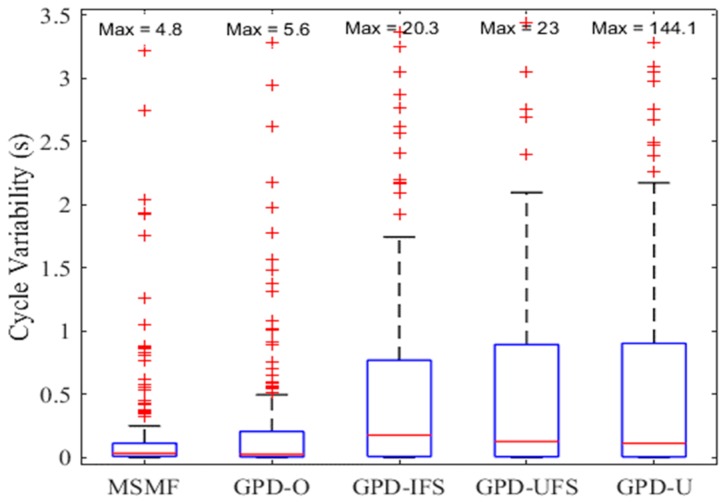
Stride variability changes with change in threshold value.

As a starting point, stride variability was investigated as a raw, unsupervised measure of an algorithms ability to accurately locate strides. Figure 8 shows a box plot of cycle variability for the MSMF and GPD algorithms. It can be seen that MSMF and GPD-O provided significantly lower cycle variability than the GPD-IFS, GPD-UFS and GPD-U approaches (*p* < 0.001, *p* < 0.001, *p* = 0.007, respectively). MSMF also trended towards a lower variability than GPD-O, although not significantly (*p* = 0.06). It should again be noted that the GPD-O is impractical for real world scenarios as the threshold needed to segment a given stride requires knowledge of the terrain. Assuming that classification of the terrain first requires segmentation of the gait cycle, this results in a circular argument. 

### 4.2. Stride Classification

**Figure 9:**
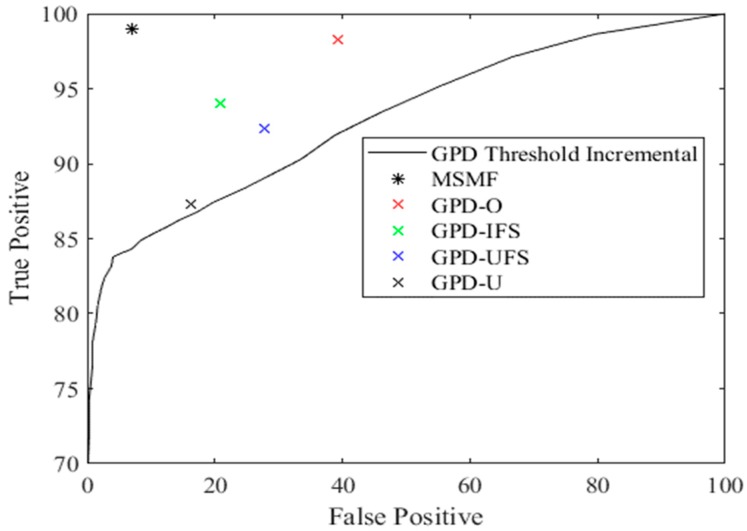
Correctly classified vs. false classified strides for various conditions. The solid line represent the GDP results obtained by changing the threshold value from 80% to 20% of the peak value.

The high levels of variability of the GPD algorithms could also in part be due to higher rates of false negative or false positive identification of strides. Figure 9 therefore shows a receiver operating characteristic (ROC) curve of the true positive and false positive rates for stride classification. Similar to the stride variability results MSMF significantly outperformed the GPD-based algorithms in both categories. Only GPD-O showed no significant difference with MSMF in true positive rates. The results of the paired sample t-tests between the MSMF and each other algorithm are shown in Table 3.

### 4.3. Algorithm Precision

**Figure 10:**
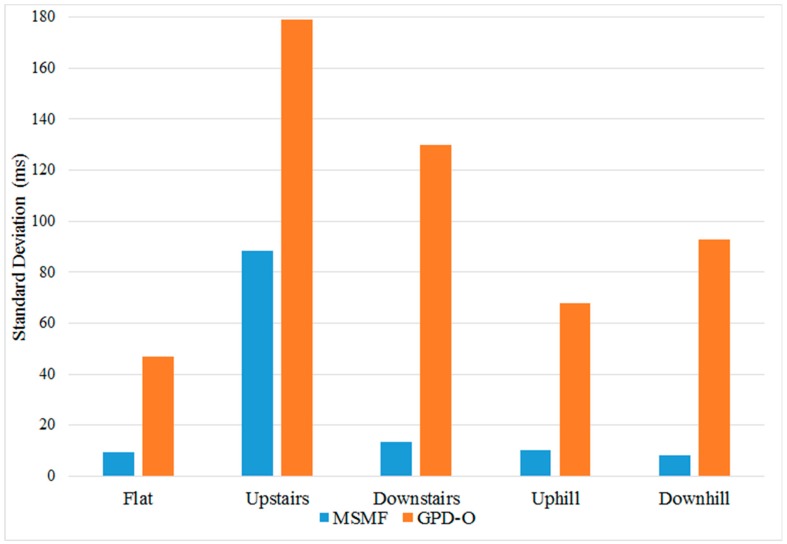
Variation of IC timing error for the MSMF and GPD-O algorithms for different terrains. Timing error is calculated as the difference in time for IC event identified by the algorithms and the push-switch.

**Figure 11:**
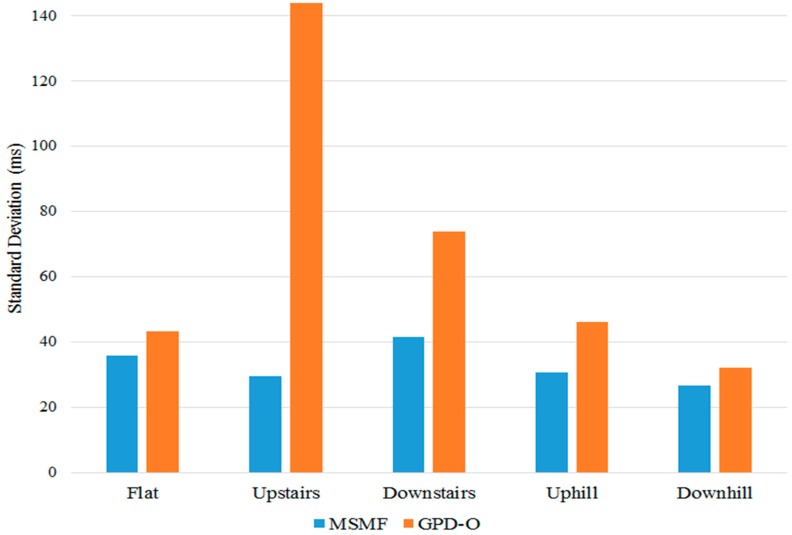
Variation of TC timing error for the MSMF and GPD-O algorithms for different terrains. Timing error is calculated as the difference in time for TC event identified by the algorithms and the push-switch.

To further compare the precision of the MSMF and GPD algorithms in detecting the IC and TC gait events, measures of timing error were computed using the push-switch pilot data. For the GPD algorithms, only GPD-O was used because it provided the maximum number of true positive results (i.e., least missed strides), yielding a large sample size with which to compare with MSMF. Timing error was defined as the difference in time between IC and TC event times, in milliseconds, identified by the algorithms and the push-switch. Figure 10 and Figure 11 show the variation of timing error for IC and TC events respectively for the different terrains. It can be seen that the MSMF algorithm consistently results in smaller error variations for IC and TC events as compared to GPD-O algorithm.

Table 4 and Table 5 show the mean and standard deviation of timing error for the MSMF and GPD-O algorithms for IC and TC events for the different terrains. Positive mean timing error values for IC indicate that, on average, the algorithm is detecting IC events before the push-switch is fully closed. Conversely, negative mean timing error in TC indicates that the algorithm is delayed in detecting TC events after the push switch has fully opened. The standard deviation of timing error indicates the variability around the mean timing error value. 

Across all terrains, it can be seen that the mean timing error in IC of the MSMF algorithm is always positive, whereas the GPD-O remains consistently negative for flat terrains, uphill and downhill walking. On stairs, however, the GPD-O displays positive values for both up- and down- stairs walking. The mean timing error for the MSMF algorithm is also smaller than that of the GPD-O for all terrains, except in up-stairs walk.

The mean timing error for TC events were always negative for the MSMF algorithm, and always positive for the GPD-O algorithm, except for the up-stairs walking terrain. Again, mean timing error values for the MSMF algorithm were generally smaller than those of the GDP-O algorithm, except for flat surface and downstairs walk.

## 5. Discussion

Alleviating the growing demands on healthcare will require new and innovative techniques and a shift towards proactive intervention. Instrumenting existing assistive devices that are already accepted may be an effective method of driving adoption of monitoring devices. In order to extract reliable and meaningful information from these devices, however, robust segmentation algorithms are needed. Accurate gait segmentation not only provides a more precise depiction of overall activity levels but is critical for subsequent analyses that rely on specific sub-elements of each stride. Both activity levels and quality of gait are vital information for the preventative monitoring of an individual’s well-being, especially for effects related to aging, such as fall prediction.

In this work, the proposed MSMF algorithm consistently demonstrated a significantly lower stride variability than even the optimized versions of the GPD algorithm, often considered a gold-standard for IMU based gait segmentation. Mean stride variance results for the MSMF algorithm were significantly better than all other GDP-based results, and trended better, but not significantly so, than those of the GDP-O. It should again be noted that the GDP-O algorithm, however, is not practical in real-world applications given its nature of simultaneously requiring both the segmented stride and type of terrain. 

The multi-sensor matched filtered approach proposed here allows for a standardized universal approach that is robust across individuals and walking terrains. The reduction in observed stride variability suggests that it provides a more representative segmentation of the individuals’ true gait. This allows for a more sensitive and accurate stride representation when perturbed gait occurs. The improved ability to analyze strides for perturbation in gait and levels of activity strengthens the case for instrumented AD’s as viable proactive monitoring tools.

The MSMF algorithm also demonstrated the highest accuracy for recognizing and determining segmented strides. A more accurate representation of a person’s walking activity is therefore being captured; a fact that is often underrated in significance. As a diagnostic tool, an improved accuracy of strides means less that the device is likely to miss strides that may communicate pertinent information about the person’s condition or level of pain. The added robustness of the system means that it is able to make these determinations across all different types of walking terrains. This allows for even greater impact as the system can be used as a normal AD in real-world environments, not just in controlled indoor clinical or lab situations. 

To further validate the algorithm, an additional pilot study was conducted with a push-switch added to the instrumented cane. Instrumenting the push-switch onto the tip of the cane provided a redundant binary measure of validation of the exact time of contact with the ground. The responsiveness of the push-switch required that 10 lbs of load be applied to the cane before a close reading could be registered. Similarly, terminal contact was recorded on the push switch once less than 10 lbs were detected. The findings show that the MSMF algorithm was not only capable of identifying the same strides as the push switch, but that it is capable of determining initial contact even *before* the 10 lbs of load is applied. Thus, it can be interpreted that the MSMF approach, when used in conjunction with an IMU and strain gauges, is in fact a more responsive method of stride representation and determination during cane assisted gait. Given the generality of the approach, it is likely that the method could be extended to work with any other system that leverages loading and motion information.

Overall, the proposed multi-sensor matched filter approach provides a robust determination of stride events over a variety of walking terrains. It has been shown to out-perform the gold-standard, even when compared to the most accurate and unrealistically ideal case. The high accuracy and robustness to various terrains enabled by the MSMF approach strengthens the case for instrumented ADs as a deployable tool that could provide valuable information and feedback to the user and their caregivers.

This is advantageous not only from an implementation standpoint, but it boosts its value as a diagnostic tool. In particular, individuals experiencing the effects of neurological injury such as PD could benefit greatly from improved methods of gait monitoring [12,51]. The high accuracy and robustness of the proposed approach strengthens the case for such ADs as deployable tools that could provide relevant information and feedback to the individual and their caregivers. Ongoing subsequent work is seeking to extend the testing of these devices to clinical populations to evaluate their ability to handle greater uncertainty, such as obstacle avoidance and turning, and validate their effectiveness as diagnostic and monitoring devices. 

## 6. Conclusions

In this paper, a robust and accurate multi-sensor matched filter-based technique for gait segmentation was proposed. Results demonstrated the ability of this novel approach to reliably and accurately segment gait cycles across a variety of different walking terrains, both indoor and outside. Specifically, it was shown that the algorithm was able to identify IC and TC gait events much more precisely than gyroscope amplitude-based segmentation approaches under different walking terrains, without the need for subject-specific thresholding. With advancements in wearable and mobile technology, such as the emergence of multi-sensor pressure sensing insoles, this algorithm may be extended to other wearable gait monitoring technologies. 

## Figures and Tables

**Table 1:** Various terrain scenarios tested for each participant.

Condition	Detail
Flat Surface	52 m on a flat surface
Upstairs	up 1 flight of stairs (20 steps)
Downstairs	down 1 flight of stairs (20 steps)
Flat-Upstairs-Flat	26 m on a flat surface, up 1 flight of stairs (20 steps), and another 26 m on a flat surface
Flat-Downstairs-Flat	26 m on a flat surface, down 1 flight of stairs (20 steps), and another 26 m on a flat surface
Uphill	78 m on a paved sidewalk, uphill
Downhill	78 m on a paved sidewalk, downhill

**Table 2:** The algorithm variations tested for each participant.

Abbreviation	Detail
MSMF	The proposed multi-sensor matched filter algorithm
GPD	Gyroscope peak detection algorithm
GPD-O	Gyroscope peak detection algorithm (a specific optimal threshold selected per participant and per terrain)
GPD-IFS	Gyroscope peak detection algorithm (individual flat surface threshold—an optimal threshold calculated specifically from a flat surface walk for each individual participant)
GPD-UFS	Gyroscope peak detection algorithm (universal flat surface threshold—a mean threshold from flat surface walking across all participants)
GPD-U	Gyroscope peak detection algorithm (universal threshold—a single mean threshold from all terrains from all participants)

**Table 3:** Comparison of true positive and false positive strides between the MSMF and GPD algorithms (paired sample t-test).

	True Positive	False Positive
MSMF vs. GPD-O	*p* = 0.4	*p* < 0.001
MSMF vs. GPD-IFS	*p* < 0.001	*p* < 0.001
MSMF vs. GPD-UFS	*p* < 0.001	*p* < 0.001
MSMF vs. GPD-U	*p* < 0.001	*p* < 0.001

**Table 4:** Timing error in IC event for MSMF and GPD-O algorithms across different terrains (with respect to push switch).

	Flat Surface (Mean ± SD)	Upstairs (Mean ± SD)	Downstairs (Mean ± SD)	Uphill (Mean ± SD)	Downhill (Mean ± SD)
MSMF	10.1 ± 9.2	72.1 ± 88.3	36.7 ± 13.5	8.8 ± 10.3	11.4 ± 8
GPD-O	−41.6 ± 46.8	32.1 ± 179.1	99.7 ± 129.8	−38.9 ± 67.9	−34.9 ± 92.9

**Table 5:** Timing error in TC event for MSMF and GPD-O algorithms across different terrains (with respect to push switch).

	Flat Surface (mean ± SD)	Upstairs (mean ± SD)	Downstairs (mean ± SD)	Uphill (mean ± SD)	Downhill (mean ± SD)
MSMF	−45.2 ± 35.7	−9.3 ± 29.5	−71.2 ± 41.6	−20.6 ± 30.7	−35.6 ± 26.7
GPD-O	42.2 ± 43.2	−108.8 ± 144	1.92 ± 73.7	37.7 ± 46.1	64.7 ± 32
